# Compensate a little, but punish a lot: Asymmetric routes to restoring justice

**DOI:** 10.1371/journal.pone.0210676

**Published:** 2019-01-10

**Authors:** Jeff Galak, Rosalind M. Chow

**Affiliations:** Carnegie Mellon University, Pittsburgh, PA, United States of America; Universidad Loyola Andalucia, SPAIN

## Abstract

Most people have a desire to live in a just world, a place where good things happen to good people and bad things happen to bad people. And yet, injustices do occur: good things happen to bad people and bad things happen to good people. Across four experiments, we show that people respond quite differently to correct these two types of injustices. When bad things happen to good people, individuals are eager to compensate a good person’s losses, but only do so to a small degree. In contrast, when a good thing happens to a bad person, because the only perceived appropriate act of punishment is to fully strip the bad actor of all his or her illegitimate gains, few people choose to punish in this costly way. However, when they do, they do so to very large degrees. Moreover, we demonstrate that differential psychological mechanisms drive this asymmetry.

## Introduction

In life, there are numerous ways in which injustice occurs. For example, it is sometimes the case that deserving individuals are passed over for employment or promotion due to systemic bias, or that seemingly guilty corporate leaders are not prosecuted due to flaws in our legal system, and instead receive generous severance packages. When injustices such as these occur, they run counter to most people’s conception of a just world, in which good things should happen to good people and bad things should happen to bad people [1, 2]. The provided examples, then, represent two classes of injustices in which outcomes have occurred to individuals who, presumably, are ill deserving of the consequences of said outcomes [3, 4]: bad events that occur to good people, and good events that occur to bad people. In this research, we ask whether the specific type of injustice–positive events befalling a bad actor or negative events befalling a good actor—affects how people will choose to restore justice, and why. When a deserving employee is passed over for promotion, what constitutes appropriate action to restore justice to his/her situation? Is it symmetrical to the action necessary to restore justice in a situation when an undeserving employee is promoted? Put differently, is the compensation necessary to restore justice when a negative outcome has occurred to a good person the same as the amount of punishment necessary to restore justice when a positive outcome has occurred to a bad person? If it is not, why are they not the same?

### The restoration of justice: Compensatory vs. retributive justice

Research on belief in a just world [2] suggests that people will act in a variety of justice-restorative behavior in response to perceived injustices [5]. For instance, individuals give quite readily to compensate innocent victims of crime [6]. People also are quite willing to punish actors who violate social norms [7, 8]. Thus, it appears that compensation of good actors for unjustly negative events is as likely as punishment of bad actors for unjustly positive outcomes; just as people are willing to incur personal cost to compensate a victim, they are also willing to incur personal cost to punish transgressors [9–11]. Moreover, individuals’ propensity to engage in either compensatory or retributive justice–compensating victims vs. punishing offenders–appears to be stronger for punishment [12, 13].

The present work departs from this prior work in two important ways. First, research comparing individuals’ preferences for compensation vs. punishment has largely focused on injustices in which the causal relationship between an actor and his/her outcome are not equivalent. For example, participants were asked how much financial repayment a transgressor (e.g., a pickpocket) should give back to a victim (e.g., the victim of a pickpocket), and the repayment was framed as either compensation for the victim or punishment for the transgressor [13, 14]. In another example, participants were asked how likely they were to vote for political candidates who had responded to injustice by compensating innocent victims (e.g., the Darfur crisis, campus bike theft), punishing perpetrators (e.g., the soldiers involved in the Darfur conflict, the bike thief), or both [12]. In both of these paradigms, which are quite typical, the victim has not caused his/her negative outcome, but the transgressor caused his/her positive outcome.

Prior work on responses to organizational (in)justice have also compared individuals’ propensity to reward good behavior vs. punish bad behavior. That is, the ability to restore justice is not constrained between the willingness to compensate victims vs. punish transgressors, as was the case in the research discussed above, but between the willingness to reward good actors vs. punish bad actors [15]. As in the studies discussed above, punishment is stronger from those who observe a bad actor’s actions than reward from those who observe a good actor’s actions. Yet, in this case too, the cases are not completely symmetrical; a good actor is to be rewarded for good behavior (no injustice has occurred) whereas a bad actor is to be punished for bad behavior (injustice has occurred). Similarly, work on social dilemmas such as public goods games has explored how punishing such free-riders vs. rewarding good contributors differentially influences group behavior and performance [16–18]. Yet this work also makes direct comparison of the two types of injustices difficult because good actors are both benefiting (by contributing to the social good) and being harmed (by being taken advantage of by free-riders) simultaneously, while bad actors are only benefited (by free-riding). Moreover, the injustice is being done *to* the good actors but is being done *by* the bad actor, making an understanding of justice is to be restored when all such variables are equated difficult. We bring up this research not to denigrate it; on the contrary, we believe that simultaneously exploring compensatory and punitive decisions is valuable (indeed, we do so here as well). But we wish to point out that in this work, the injustices studied and paradigms used do not allow for exact comparisons between compensatory and punitive responses to injustice because the injustices involved are not of equivalent types; either the researchers compare compensation for innocent victims vs. punishment of guilty transgressors, which are not causally equivalent, or they compare reward for good/just behavior vs. punishment for bad/unjust behavior.

The present work attempts to remedy this empirical omission by focusing on injustices where the outcomes are completely incidental to the behaviors of the actors, making both types of injustice completely symmetric in nature. We do so because our interest is specifically in responses to outcomes that people will admit to being apparently random, and yet, still see as unjust. By specifying that there is no causal link between actor and consequence, we can avoid issues related to differential attributions of why outcomes should occur to good or bad people [19]. In so doing, we aim to expand our understanding of the relative thresholds individuals believe punishment and compensation must achieve for justice to be restored for objectively equivalent injustices–but of different types: positive outcomes for bad actors and negative outcomes for good actors—and additionally investigate the determinants of these different thresholds.

If outcomes are obtained through random chance, why would people consider those outcomes to be unjust? Prior work on immanent justice suggests that people will still respond as if the beneficiary or victim is personally responsible for those outcomes even if the outcomes are due to random chance; people attribute deservingness of positive or negative outcomes to individuals as a function of how good or bad a person they are, even when no obvious causal link exists between moral character and outcomes [20, 21]. Consider, for example, a case of an employee known to bully other workers and who randomly wins the lottery; even though it is obvious that his bullying behavior did not cause him to win, it is likely his co-workers would still consider it unfair that such an abusive individual should win the lottery. Thus, even if a person has not directly caused his/her outcome, it is likely that individuals still perceive the outcome to be unjust and will wish to rectify the situation. The question, then, is whether individuals differ in their beliefs about what it would take to restore justice to a situation where a transgressor obtained a positive outcome, but through no action of his/her own, as compared to a situation where a victim obtained a negative outcome, but through no action of his/her own.

We predict that in a situation where a bad event befalls a good person, the threshold for what it takes to restore justice is relatively low, as compared to the threshold for restoring justice when a good event befalls a bad person. We contend that this is the case because although there is a general belief that good actors ought to be rewarded, resulting in greater frequency of compensating victims, good behavior is also considered to be normative, thus resulting in lower amounts of compensation [15, 22]. That is, because good behavior is normal, being good does not entitle one to rewards that are especially generous. Thus, to restore justice to a good person who has obtained a negative outcome, people give willingly (i.e., with high frequency), but small amounts are sufficient to fulfill their obligation (i.e., in small quantities). Consistent with this possibility, people are relatively scope insensitive to the amount of compensation they provide in terms of their assessment of how much good they have done; it is sufficient that something was given, regardless of amount, for people to feel as though they have done good [23]. In addition, people readily give help to victims of natural disasters, but they do so individually to small degrees [24]. Moreover, they generally feel just as morally good regardless of the amount they have spent [25]. Taken together, these results suggest that people will be more willing to help innocent victims (frequency), but only to small degrees (amount), and that this lower threshold for amount may be due to the lower threshold for what people believe will fulfill their moral obligation for rewarding good behavior (see [15] for a similar prediction and finding for third-party observers).

In contrast, we propose that in a situation where a good event befalls a bad person, there is a relatively high threshold for justice-restoration. Importantly, the threshold, in this instance, is tied not to fulfilling a personal moral obligation to help victims [26, 27], as is the case for compensation of innocent victims, but is driven by a desire to maintain social order and to deter future negative behavior (i.e., a social moral obligation, [15, 28]). To the extent that punishment is meant to act as a deterrent to future bad behavior [29–31], and is, in fact, effective at increasing cooperation in groups [32] (though see [33] for an important exception), small punishments will be seen as ineffective [34]. Indeed, if deterrence is the goal, the most effective punishment is extreme sanctioning [35, 36], which signals that the behavior in question is morally unacceptable [37, 38]. Notably, complete and severe punishment is often quite costly for the enforcer, perhaps accounting for the relatively low frequencies of punishment in prior work [11]. In sum, it is our contention that although the overall frequency of punishment will be low in response to a situation where a bad person has a positive outcome, when people do choose to punish in order to restore justice, it will be for a large amount due to their underlying desire to deter future bad behavior. It is worth noting that previous research has also documented instances of punishment behavior that is purely retributive, as opposed to deterrent focused [39–41]. That is, sometimes people want to punish just to be vengeful, and not to prevent future bad behavior. Though this motivation likely exists is present within our research paradigm, in our final experiment, we directly assess deterrent motivations and observe that they are the primary driver for punishing bad actors who benefited from a positive event.

To summarize, our primary contention is that justice-restoration is satisfied by giving a small amount to compensate for illegitimate losses, but is not satisfied unless illegitimate gains are completely removed, leading to larger amounts of punishment. These differences should be associated with differing thresholds for what counts for having fulfilled one’s moral obligation to victims and what will be effective at deterring future bad behavior, respectively. Across all studies, sample sizes were arbitrarily determined prior to data collection, and exclusions are reported. Data collection was terminated when the sample size closely approached or reached the target and analysis occurred only after data collection was terminated. We report all independent and dependent variables collected. Of note, prior work has documented moral outrage as a driver of justice-restorative behavior [7, 42–44]. In our S2 Appendix, we report the results of a separate study in which we find differences in emotional responses, but these differences do not account for participants’ justice-restorative behavior.

## Study 1

To test our predictions, we used a third-party observer dictator game [9] involving what participants believed were two individuals randomly assigned to be a decider or a receiver (actually computer simulations), and the third (the participant) to be an observer. The decider unilaterally decides how much of a fixed sum of money to share with the receiver. Prior work using this paradigm has shown that deciders who give roughly half of their allocation to the receiver are seen as good people, whereas deciders who keep most or all of the allocation are seen as bad people [45, 46]. We therefore manipulated the goodness/badness of an actor by varying the amount of the decider’s allocation to the receiver.

Specifically, half of the participants (the observers) saw that the decider kept all the money across five rounds of play (acting badly), whereas the other half observed that the decider shared exactly half of the money across all five rounds (acting justly), giving half to the receiver. The decider then received an exogenous harm or windfall, such that the “bad” decider who kept all the money for him or herself was “randomly” rewarded with a bonus sum of money, a good event. The “good” decider who shared his or her money was instead “randomly” punished with a penalty removing some of his or her money, a bad event. In this way, the event was due to an obviously random external mechanism. Finally, to allow participants to either punish the bad actor or compensate the good actor, they could, at personal cost, spend money to either take money from the bad actor or give money to the good actor. This experimental paradigm allowed us to examine what actions people take to restore justice when faced with a bad actor illegitimately benefiting or a good actor unfairly suffering.

Of note, we chose to use a deception based study for two key reasons: 1) cost of administration is far lower and, more critically, 2) the study paradigms employed all require very specific behaviors from the decider (computer simulation in our case). Allowing the decider to be a real person would make it nearly impossible to obtain the sample sizes needed to actually test the hypotheses we are interested in. The only significant benefit to including all real participants, as opposed to some computer simulations that appear to be real participants, would be to observe what distribution of choices deciders make, but that is in no way relevant to our research questions. Instead, we only seek to understand how observes respond to deciders who choose to act fairly or selfishly. By using computer simulations to act in a fixed way for all study participants we are thus able to directly examine the responses of the observers while keeping the “behavior” of the deciders constant.

### Participants

400 participants (Median Age = 31; 49.9% female, 49.9% male, 0.2% unknown gender) were recruited from the Amazon Mechanical Turk (mTurk) online panel and paid a $1.00 show up fee regardless of their actions during the study. Of those, three started the study, but never reached any of the dependent measures. Of the remaining 397, 12 sets of responses came from participants who shared the same IP address (e.g. two completions from a single IP address). To avoid the possibility that individuals completed the study more than once, all such completions were excluded from analyses. This resulted in usable data from 385 participants. Of note, across all of our studies, results do not meaningfully differ if exclusions are omitted.

### Procedure

Participants were recruited to participate in what they believed was a 3-player study. In fact, participants completed the study alone and the other two players in the study were computer simulations designed to appear as if they were real human players. Participants were not informed that they were actually playing alone and all efforts were made to convince participants that other human players were involved (more on this below). The Carnegie Mellon University institutional review board reviewed and approved all research conducted within this paper (ID#IRB_STUDY2015_00000053). In all cases, written consent was collected using an online form approved by the same review board.

**Fig 1:**
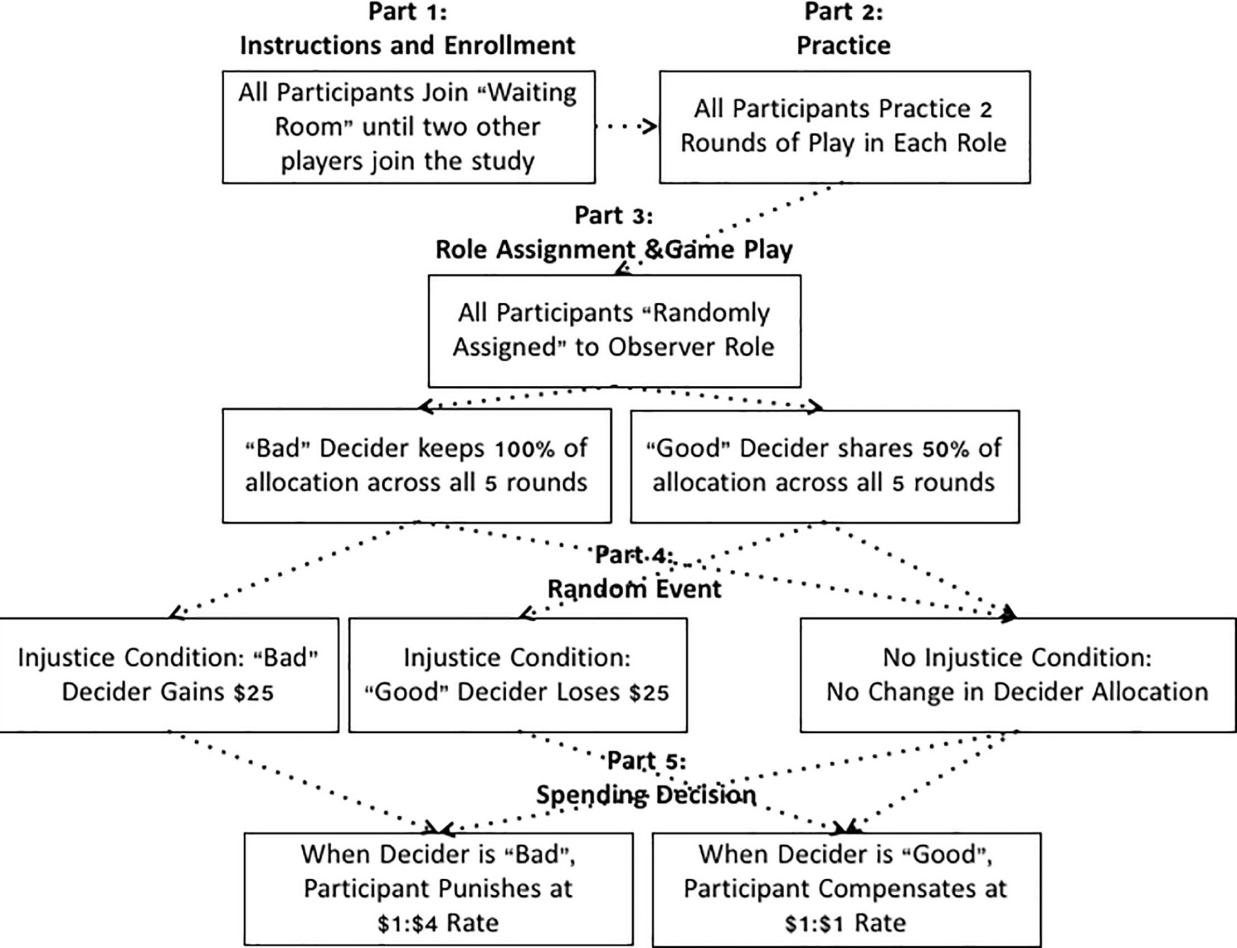
Summary of game parts.

The study consisted of five parts. Accordingly, for simplicity, we describe the procedure for each part separately, but, for participants, there were no such categorical components to their experience. A graphical summary of the experimental procedure can be seen in Fig 1.

#### Part 1 –instructions and enrollment

Participants were informed that they would be participating in a “decision game” with two other players. To bolster their belief that there were two other human players, they were told that they would first join a “waiting room” to wait for the other two players to arrive (see Figure A in S1 Appendix). They were told that if, after 5 minutes, the other two players did not arrive, they would be paid their show up fee and would not be required to complete any other portions of the study. Once they read these instructions, participants saw a screen titled “Waiting Room” with a small box in the upper right corner summarizing the current players in the game. “Player 1” was already in the game and they joined as “Player 2 (you)”. Fourteen seconds after joining the “Waiting Room,” “Player 3” joined the game and appeared in the same box. The program then informed the participant that all players have joined and that the game would begin shortly. Three seconds later, participants were taken to the next screen.

At this point, participants read a detailed description of the upcoming game. They were told that the program would randomly assign each player a role: a Decider, a Receiver, and an Observer. The game would consist of 5 rounds where the Decider would be allocated $20 each round and could split that allocation with the Receiver in any way he or she wanted. The Observer would be paid a fixed fee of $10 per round regardless of the Decider’s actions. They were honestly informed that one group of participants would be chosen at random to receive a bonus payment based on the decisions in the game (one participant was chosen at random from each experiment to be paid the bonus based on his or her actions during the corresponding study). This served to make their decisions incentive compatible. They were also informed that they would first complete 6 practice rounds taking on each role twice in order to familiarize all players with the mechanics of the game.

Once players read the instructions and clicked the “continue” button, a loading spinner animation and the text “Please wait for all players to hit ‘continue’” appeared on this screen. This animation and text appeared on every screen in the study and remained on screen for a randomly predetermined amount of time ranging from 3 to 20 seconds. This was designed to increase the believability that other human players were involved.

#### Part 2: Practice

Following the instructions, participants completed 6 practice rounds of play. They first took on the role of the Receiver and, for Round 1, witnessed that the Decider chose to keep $10 for him/herself and give $10 to the Receiver (the participants in this case; see Figure B in S1 Appendix). During Practice Round 2, the Decider kept $12 and gave $8 to the Receiver (the participant). During the next 2 practice rounds, the participant took on the role of the Decider and chose any allocation they wanted (see Figure C in S1 Appendix). During the final two practice rounds, the participant took on the role of the Observer. In both rounds 5 and 6, the Decider kept $10 for him/herself and gave $10 to the Receiver. These practice rounds were designed to both familiarize the participant with the nature of the game and to increase the believability that there were two other human players. Moreover, that the other players always gave money to the Receiver served as a way to reinforce that sharing the payment was normative.

#### Part 3: Role assignment and game play

Following the practice rounds, participants were informed that the program would now randomly assign them to the roles that they would play during the main part of the study. To increase believability, the program displayed an animation where the roles for each player quickly changed as if being randomly determined. The program settled on roles such that Player 1 was the Decider, Player 2 (participant) was the Observer, and Player 3 was the Receiver. The participant was then reminded that, as the Observer, he or she would be paid $10 per round regardless of what the Decider did. Next, the participant acted as an Observer for 5 rounds. During each round, the program randomly varied the amount of time the Decider took to make a decision between 6 and 20 seconds (between 15 and 20 seconds for Round 1 as the first round is likely to take the longest for real human players). At this point, participants were randomly assigned to one of two *Person Type* conditions. In the *Bad Person* condition, the Decider, on all 5 rounds, acted selfishly and kept the entire $20 for him/herself. In contrast, in the *Good Person* condition, the Decider, on all 5 rounds, acted fairly and split his/her $20 evenly between him/herself and the Receiver. A summary of decisions and payouts is reported in Table 1.

**Table 1:** Study 1 summary of actions and payouts.

Injustice Presence	Person Type	Decider Action	Payouts Following Game Play			Payouts Following Random Event		
			Decider	Receiver	Observer	Decider	Receiver	Observer
Injustice	Good	Split Allocation 50/50	$50	$50	$50	$25	$50	$50
	Bad	Keep Entire Allocation	$100	$0	$50	$125	$0	$50
No Injustice	Good	Split Allocation 50/50	$50	$50	$50	-	-	-
	Bad	Keep Entire Allocation	$100	$0	$50	-	-	-

Following all 5 rounds, as a manipulation check, all participants answered the following question: “How would you characterize Player 1 (Decider)?” on a 9-point scale anchored with -4 (Extremely Bad Person), 0 (Neutral), and +4 (Extremely Good Person).

#### Part 4: Random event

One goal of this study was to ascertain if people simply respond to good versus bad actions, rather than good versus bad events that befall bad versus good people. In other words, any decision to compensate or punish may be due to the actions that the Decider took and not due to independent events that befell them. In order to test this, participants were randomly assigned to one of two *Injustice Presence* conditions. For participants in the *Injustice* condition, they read that there was an additional game phase in the experiment. Participants in the *Bad Person* condition were told that one player in the game would be randomly assigned to earn an extra $25. In contrast, participants in the *Good Person* condition were told that one player in the game would be randomly assigned to lose $25 of their earnings. In all cases, the computer “randomly” selected the Decider to either receive the bonus $25 (if they were a *Bad Person*) or to lose the $25 (if they were a *Good Person*, see Figure D in S1 Appendix). In this way, the bad person received a presumably unjust windfall, while the good person lost a presumably unjust sum of money. For participants randomly assigned to the *No Injustice* condition, no such random gains or losses took place–they only witnessed a Decider who either chose to keep all the monies for him/herself or a Decider who chose to share the monies equally with the Receiver.

#### Part 5: Spending decision

All participants were next told that there was one final decision to be made during this study. Participants in the *Bad Person* condition were told that they could spend any of their non-show-up-fee earnings ($50) to take money away from the Decider (who, at this point, had either $100 in the *No Injustice* condition or $125 in the *Injustice* condition). For every $1 they spent, $4 would be taken away from the Decider (this multiplier was applied following a procedure used by [9] and to allow for the possibility of removing all of the Deciders funds (by spending $32) if the participant chose to do so). This acted as a form of punishment that could be bestowed on the Decider for being the beneficiary of an injustice (in the *Injustice* condition), or for just being a bad person (in the *No Injustice* condition). Participants in the *Good Person* condition were told that they could spend any of their non-show-up-fee earnings ($50) to give money to the Decider (who, at this point, had either $50 in the *No Injustice* condition or $25 in the *Injustice* condition). For every $1 they spent, $1 would be given to the Decider. This acted as a form of compensation that could be bestowed on the Decider for being the victim of an injustice (in the *Injustice* condition) or for just being a good person (in the *No Injustice* condition). All participants were then given a table summarizing all payouts that were originally to be given to all players and the payout that would occur based on their decision to either punish (*Bad Person*) or compensate (*Good Person*) the Decider. They then made their decision (see Figure E in S1 Appendix).

Finally, all participants indicated, in an open-ended text box, why they chose to spend what they did on the previous screen, whether or not they were familiar with the Dictator Game, whether or not they actively participated in the study, their belief about the purpose of the study, additional comments for the experimenter and some basic demographic information. Of note, neither participants’ prior knowledge of the Dictator Game nor their stated attention during the study significantly moderated any of our results and so will not be mentioned further.

### Results

#### Preliminary analyses

We first wanted to see if participants were suspicious of the design of the study and the human vs. computer nature of the other two players. To do so, all open-ended comments were read and any mention of suspicion related to the nature of the other participants was noted. Of the 385 participants, only 18 (4.7%) indicated any suspicion that the other players were not real, suggesting that our experiment successfully convinced participants that they were playing with two other humans. Moreover, analyses excluding suspicious participants did not meaningfully change our results.

We next confirm that the Decider was, in fact, perceived as being a good or bad person depending on their actions in the two *Person Type* conditions. A 2 (Person Type: Bad vs Good) x 2 (*Injustice Presence*: No Injustice vs. Injustice) ANOVA on perceived goodness/badness of the Decider yielded only a significant main effect of *Person Type* (F(1, 381) = 1481.65, p < .001, partial-η^2^ = .79; all other ps > .25). Specifically, participants perceived the Decider in the *Bad Person* condition to be particularly bad (M = -2.57; one-sample t-test vs 0: t(197) = -23.31, p < .001) whereas they perceived the Decider in the *Good Person* condition to be particularly good (M = 3.02 one-sample t-test vs 0: t(198) = 32.21, p < .001).

#### Primary decision; punish vs. compensate

We first analyze the decision to punish vs compensate by assessing differences in spending amounts across all conditions. Because spending rates are highly non-normally distributed (Shapiro-Wilk test of normality(385) = .29, p < .001), we cannot use simple parametric analyses such as ANOVA. Instead we assess differences in spending rates with non-parametric tests. Specifically, a Kruskal-Wallis test on spending rates across conditions yields a significant effect (chi-sq (3) = 61.36, p < .001), indicating that spending did vary across conditions. For presentation purposes, we report means rather than medians, but use Mann-Whitney tests for pair-wise comparisons. Specifically, we find that when the Decider was a *Good Person* (M_No Injustice_ = 2.37, M_Injustice_ = 9.64; Mann-Whitney U = 2118, Z = 6.97, p < .001) and, to a lesser degree, when the Decider was a *Bad Person* (M_No Injustice_ = 2.69, M_Injustice_ = 7.59; Mann-Whitney U = 4014.5, Z = 1.72, p = .09), participants spent more to compensate or punish when an injustice occurred as compared to when an injustice did not occur. This result suggests that participants believed that the randomly assigned loss/gain to the Deciders were true injustices that needed to be rectified, whereas regular game play (i.e. being selfish or generous) were not instances of unjust outcomes that required remedy. Of note, in aggregate, participants did not spend more or less to compensate the *Good Person* vs. punish the *Bad Person* when no injustice occurred (M_Good Person_ = 2.37, M_Bad Person_ = 2.69, Mann-Whitney U = 4868.0, Z = -0.93, p = .93), but did spend more to compensate the *Good Person* vs. punish the *Bad Person* when an injustice did occur (M_Good Person_ = 9.64, M_Bad Person_ = 7.59, Mann-Whitney U = 3445.0, Z = -3.88, p < .001). Though this may suggest that people are more willing to help good people who suffer an injustice than to punish bad people who benefit from an unearned windfall, the results are quite different when considering the question of whether participants chose to spend any amount of their own money as well as the quantity they chose to spend, conditional on spending anything.

**Fig 2:**
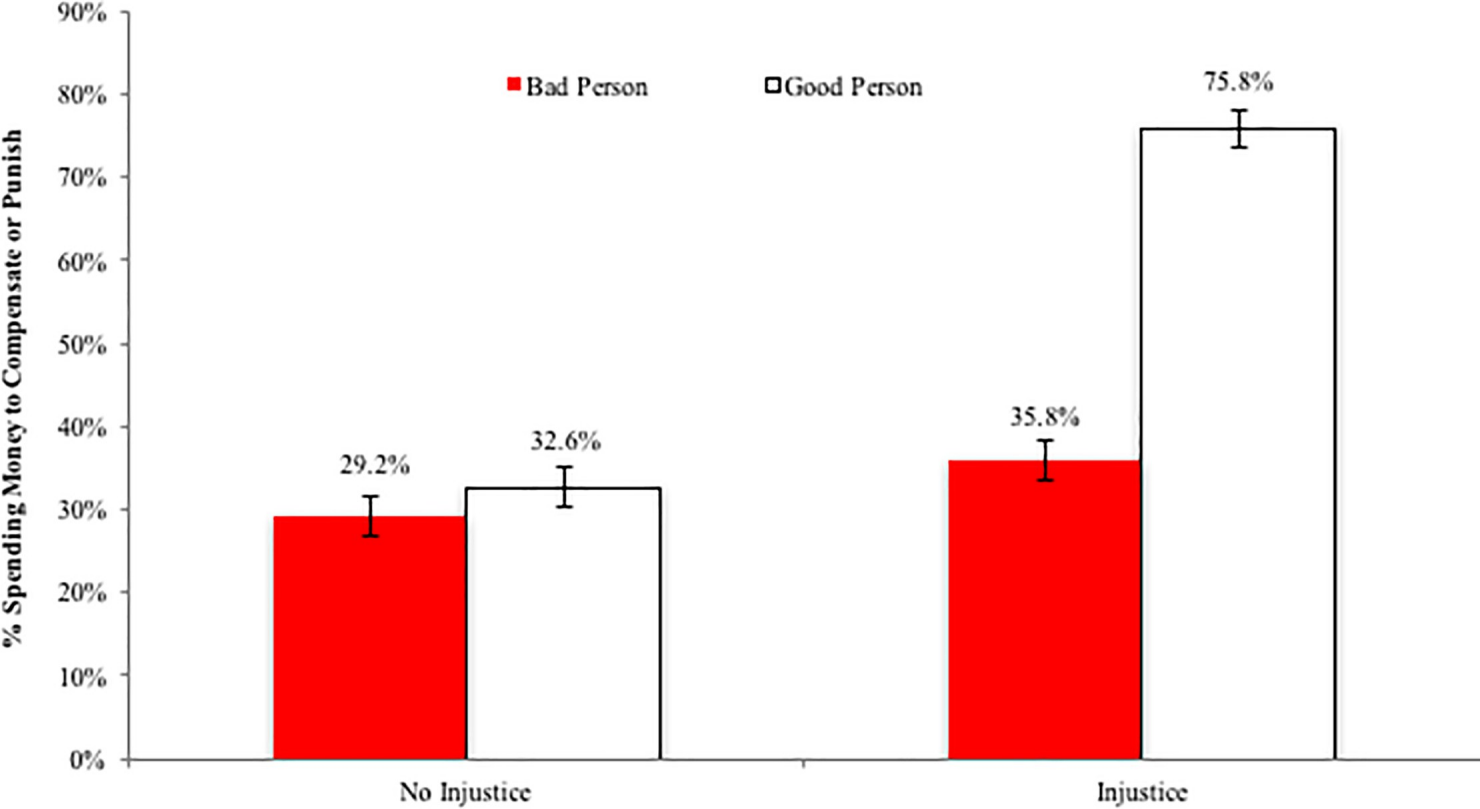
Study 1: % of participants spending anything to compensate or punish. Error bars represent standard errors.

To that end, a logistic regression predicting the likelihood of giving any amount of money as a function of the two independent variables and their interaction yielded a significant interaction (B = 1.56, SE = .45, p < .001). Specifically, as can be seen in Fig 2, whereas participants were roughly equally likely to punish a bad person (29.2%) as they were to compensate a good person (32.6%) when no injustice transpired (B = -.16, SE = .31, p = .60), they were far more likely to compensate a good person who experienced a negative event (75.8%) than they were to punish a bad person who benefited from a positive event (35.8%; B = -1.72, SE = .32, p < .001).

**Fig 3:**
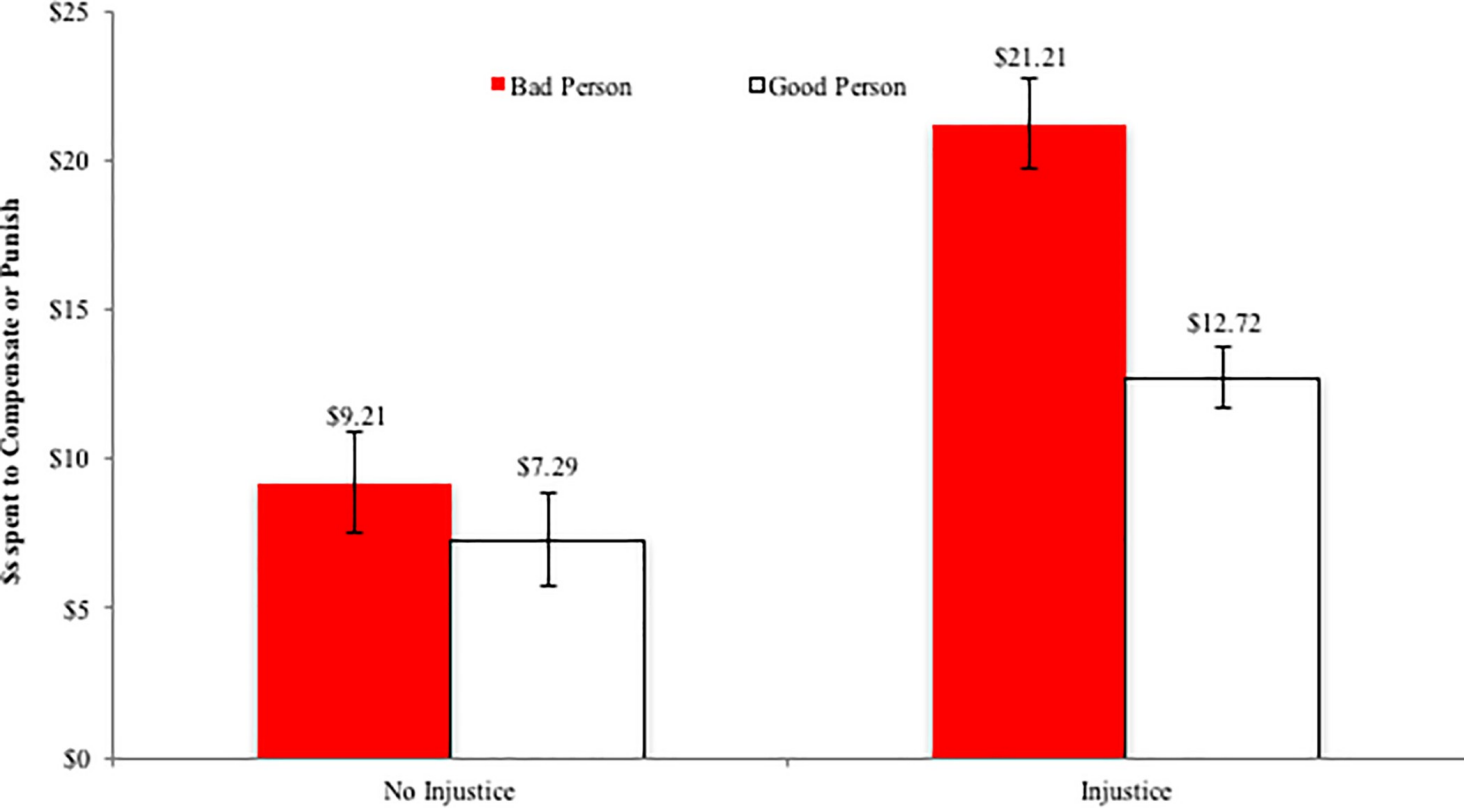
Study 1: Amount spent by those who spent anything to compensate or punish. Error bars represent standard errors.

**Fig 4:**
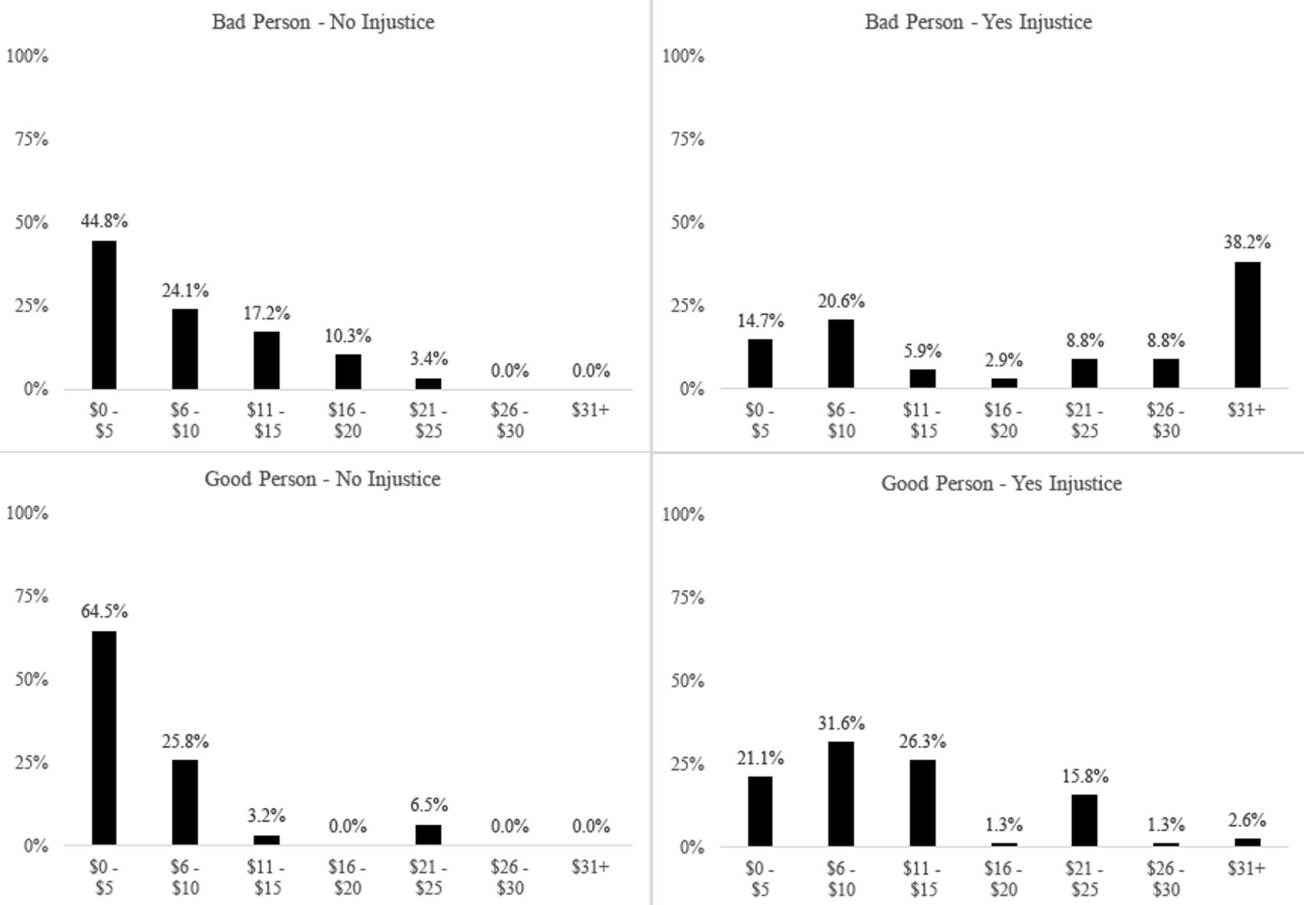
Study 1: Histogram of spending amounts by those who spent anything to compensate or punish, by condition.

However, despite the large difference in the numbers of people who spent anything to compensate or punish, the rates at which these people did so was exactly opposite. We again use non-parametric tests, but report means for simplicity. Specifically, a Kruskal-Wallis test on spending rates for those who spent anything across conditions yields a significant effect (chi-sq (3) = 10.64, p < .001), indicating that spending did vary across conditions. As can be seen in Figs 3 and 4, participants in the *No Injustice* conditions who elected to spend anything at all tended to spend more to punish the bad person than to compensate the good person (M_Bad Person_ = $9.21 vs. M_Good Person_ = $7.29; Mann-Whitney U = 319, Z = -1.85, p = .06). This difference was greatly magnified in the Injustice condition; for those in the *Injustice* conditions, participants who spent any money, spent considerably more to punish a bad person who had a good action befall them (M = $21.21) as compared to compensate a good person who had a bad action befall them (M = $12.72; Mann-Whitney U = 825.5, Z = -2.96, p = .003). Indeed, the only condition under which spending rates meaningfully deviated from the other conditions is when a good thing happened to a bad person.

In sum, these findings suggest that when an injustice occurs, peoples’ preferences for justice restoration depend on the type of injustice. When a hardship befalls a good person, many people will give a small amount. Indeed, the median amount spent by those who chose to compensate the good person who suffered an injustice was $10, only 1/5^th^ of their own allocation. To that end, many people are willing to spend their own money to help a victim, because even small amounts are likely seen as justice restorative. In contrast, when a bad person benefits from an unjust windfall, punishment needs to be far greater to restore justice, which leads to lower overall likelihood of punishment, but larger amounts of punishment when punishment is given. Specifically, even though the percentage of people who chose to punish the bad person were lower, the median amount spent by those who chose to punish the bad person who benefited from an injustice was $25, half of their own allocation and enough to remove nearly all ($100 of $125) of the Decider’s money. Moreover, 26.5% of those who chose to punish the bad person benefiting from an injustice chose to spend at least $32, the minimum needed to take all of the Decider’s money away.

Though this study supports our key hypotheses, there are at least two possible alternative explanations for these results. First, it is possible that the key driver for Observers across all conditions was some form of inequality aversion, or the desire to equate the final sum of funds that they possessed and the Decider possessed [47], rather than to restore justice in the way we outlined above. That is, participants may have chosen to spend precisely the amount of money needed to ensure that the Decider had no more or no less than they did, and this may have been true in all conditions. For instance, spending about $12 in the *Good Person Injustice* condition would result in the Decider having $37 ($25 + $12) and the Observer having $38 ($50 - $12). Likewise, spending $25 in the *Bad Person Injustice* condition would result in the Decider having $25 ($125–4 x $25) and the Observer having $25 ($50 - $25). If so, such a difference in spending amounts ($12 in the *Good Person Injustice* condition versus $25 in the *Bad Person Injustice* condition) could be fully explained by a desire to equate the amount of funds between the Observer and the Decider rather than a desire to provide either some small relief to the good person or (near) total punishment to the bad person.

A second alternative is that the relatively low rate of spending in the *Bad Person Injustice* condition may be driven by Observers’ concern that the punishment was too severe. That is, taking $4 away from the bad Decider for every $1 spent may have seemed too extreme a response. To address these two possible alternative accounts, we conducted two conceptual replications of the present study with the direct purpose of ruling them out.

## Study 2a

The purpose of Study 2a is to rule out the possibility that participants chose their spending amounts in Study 1 to equalize their payouts with that of the Decider. To that end, we replicate Study 1, but systematically vary the payout structure of the Observer to allow us to test whether equality considerations played a major role in determining the amount spent by Observers.

### Participants

One hundred ninety-nine participants (Median Age = 33; 50.8% female, 48.7% male, 0.5% unknown gender) were recruited from the Amazon Mechanical Turk (mTurk) online panel and paid a $1.00 show up fee regardless of their actions during the study. No responses shared the same IP address (e.g., two completions from a single IP address). For this study and all subsequent studies, TurkGate software was used to ensure that participants from previous studies could not complete additional studies [48].

### Procedure

The procedure was identical to Study 1 with two key differences. First, only the two *Injustice* conditions were included, as they are the ones needed to test for an equality account of our findings. Second, and most critical, we varied the payouts to the Observer across the two *Injustice* conditions. Specifically, Observers in the *Good Person Injustice* condition were paid $5 per round (as opposed to $10 per round, as in Study 1). This resulted in Observers ending the game with $25, rather than $50. Importantly, the Decider also ended with $25 ($50-$25 random event). As such, an equality account would predict virtually no spending by Observers, as they already have the same payout as the Decider. In contrast, our theorizing predicts that Observers will still spend some nominal amount of money to restore justice stemming from the perceived unfair removal of funds from the Decider.

Similarly, the Observers in the *Bad Person Injustice* condition were paid $20 per round (as opposed to $10 per round, as in Study 1). This resulted in Observers ending the game with $100, rather than $50. The Decider still ended the game with $125 ($125 + $25 random event). An equality account would predict that Observers would spend around $9 dollars to take away around $36 from the bad Decider, as this would result in relative equality in payouts for the Deciders ($89) and Observers ($91). In contrast, our theorizing predicts little deviation in spending behavior from what we observed in Study 1. Specifically, our theorizing predicts that Observers will still spend a large sum of money to maximally punish the Decider, even if doing so does not result in equal payouts for the Decider and the Observer.

### Results

#### Preliminary analyses

We again wanted to see if participants were suspicious of the design of the study or that they were playing against a computer rather than a person. To do so, we read all open-ended comments and noted any mention of suspicion related to the nature of the other participants. Of the 199 participants, only 7 (3.5%) indicated any suspicion that the other players were not real, suggesting that our experiment again successfully convinced participants that they were playing with two other humans. Moreover, analyses excluding suspicious participants did not meaningfully change our results.

We next confirmed that the Decider was, in fact, perceived as being a good or bad person depending on his or her actions across conditions. Replicating the results in Study 1, the bad person was seen as far worse than the good person (*t*(197) = 23.25, *p* < .001, *d* = 3.13). Specifically, participants perceived the Decider in the *Bad Person Injustice* condition to be particularly bad (M = -1.99; one-sample t-test vs. 0: t(101) = -11.13, p < .001, d = 1.10), whereas they perceived the Decider in the *Good Person* condition to be particularly good (M = 3.10 one-sample t-test vs. 0: t(96) = 25.22, p < .001, d = 2.55).

#### Primary decision; punish versus compensate

We first analyze the decision to punish versus compensate by assessing differences in overall spending amounts across conditions. As in Study 1, spending rates were highly non-normally distributed (Shapiro-Wilk test of normality (199) = .352, p < .001). Thus, we assess differences in spending rates with non-parametric tests, but report means for ease of comprehension. Specifically, unlike in Study 1, a Mann-Whitney U test on spending rates across conditions did not yield a significant effect (Z = -1.35, p = .18), indicating that spending, on average, did not vary across conditions (M_Bad Person Injustice_ = $6.93, M_Good Person Injustice_ = $3.93).

However, the results replicate those found in Study 1 when we consider the question of whether participants chose to spend any amount of their own money as well as the quantity they chose to spend, conditional on spending anything. A logistic regression predicting the likelihood of giving any amount of money as a function of injustice type yielded a significant result (B = -.845, SE = .31, p < .001). Specifically, consistent with Study 1, participants were far more likely to spend anything to compensate the Good person who randomly lost money (44.3%) than they were to punish the bad person who randomly gained money (25.5%).

More central to the purpose of this study, the spending amounts conditional on spending anything also replicated the results of Study 1. Specifically, a Mann-Whitney U test on spending rates for those who spent anything across conditions yielded a significant effect (Z = -5.86, p < .001), indicating that spending, on average, varied greatly across conditions for those who chose to spend anything (M_Bad Person Injustice_ = $27.19, M_Good Person Injustice_ = $8.86). These results are inconsistent with an equality account. That is, the desire to equate the amount received by the Decider and the Observer would have resulted in spending of about $9 in the *Bad Person Injustice* Condition and no spending in the *Good Person Injustice* condition, which is not what we found.

## Study 2b

The purpose of Study 2b was to test the second alternative explanation to the results in Study 1. Specifically, it is possible that the act of punishing a bad Decider at a rate of $4 removed for every $1 spent (4:1 ratio) was seen as too extreme, which could account for the relatively low frequency of punishment. If this is true, then if participants were given a less severe punishing scheme (e.g., $1 spent equaling $1 removed), they would do so at rates equal to those of Observers who were compensating a good Decider. In contrast, our theorizing would predict very low spending rates in the *Bad Person Injustice* condition with such a 1:1 punishment scheme, as it would not allow for extreme sanctioning (i.e., removal of all funds). To that end, we conducted this study and changed the punishment rate from 4:1 to 1:1.

Of note, this change in punishment rate also helps rule out inequality aversion and spitefulness as drivers of our results. For the former, a 1:1 punishment rate cannot change relative payoffs, and, for the latter, previous research has shown that spitefulness behavior disappears with such an ineffective punishment rate [49].

### Participants

One hundred ninety-eight participants (Median Age = 34; 52.5% female, 47.0% male, 0.5% unknown gender) were recruited from the Amazon Mechanical Turk (mTurk) online panel and paid a $1.00 show up fee regardless of their actions during the study. No responses shared the same IP address (e.g., two completions from a single IP address).

### Procedure

The procedure was identical to Study 1 with two key differences. First, again only the two *Injustice* conditions were included, as they are the ones needed to test the influence of a 1:1 punishment rate on spending behavior in the *Bad Person Injustice* condition. Second, and most critical, the rate at which Observers could remove funds from the bad person was changed to be 1:1, the same rate at which Observers could supply funds to the Good person. If punishing at a 4:1 rate (as in Study 1) was seen as so extreme that few participants chose to do so, we should expect to see much higher rates of punishment in this study. If, instead, punishment needs to be extreme to restore justice, as we predicted and observed in Study 1 and 2a, then we should see very low levels of punishment behavior, as extreme sanctioning is impossible under this punishment scheme.

### Results

#### Preliminary analyses

We again wanted to see if participants were suspicious of the design of the study or that they were playing against a computer rather than a person. To do so, we read all open-ended comments and noted any mention of suspicion related to the nature of the other participants. Of the 198 participants, only 12 (6.1%) indicated any suspicion that the other players were not real, suggesting that our experiment again successfully convinced participants that they were playing with two other humans. Moreover, analyses excluding suspicious participants did not meaningfully change our results.

We next confirmed that the Decider was, in fact, perceived as being a good or bad person depending on his or her actions across conditions. Replicating the results in Studies 1 and 2a, the bad person was seen as far worse than the good person (*t*(196) = 28.45, *p* < .001, *d* = 3.66). Specifically, participants perceived the Decider in the *Bad Person Injustice* condition to be particularly bad (M = -2.37; one-sample t-test vs. 0: t(86) = -11.90, p < .001, d = 1.28), whereas they perceived the Decider in the *Good Person* condition to be particularly good (M = 3.23 one-sample t-test vs. 0: t(110) = 30.41, p < .001, d = 2.88).

#### Primary decision; punish versus compensate

We first analyze the decision to punish versus compensate by assessing differences in overall spending amounts across conditions. Because spending rates are highly non-normally distributed (Shapiro-Wilk test of normality (198) = .296, p < .001), we assess differences in spending rates with non-parametric tests, but report means for ease of comprehension. Specifically, a Mann-Whitney U test on spending rates across conditions yielded a significant effect (Z = -7.76, p < .001, indicating that spending, on average, was far higher in the Good Person *Injustice* condition (M = $9.66) than in the *Bad Person Injustice* condition (M = $1.54).

The results replicate those found in Studies 1 and 2a when we consider the question of whether participants chose to spend any amount of their own money as well as the quantity they chose to spend, conditional on spending anything. A logistic regression predicting the likelihood of giving any amount of money as a function of injustice type yielded a significant result (B = 2.60, SE = .36, p < .001). Specifically, consistent with Studies 1 and 2a, participants were far more likely to spend anything to compensate the Good person who randomly lost money (72.1%) than they were to punish the bad person who randomly gained money (16.1%). This result, though consistent with our theorizing and past findings, is inconsistent with the notion that punishment rates would increase because a 1:1 punishment rate is seen as more acceptable than a harsher 4:1 punishment rate.

Furthermore, the spending amounts conditional on spending anything run counter to the alternative explanation being tested here, which would argue for equivalent spending amounts between the two conditions or greater spending amounts among Observers of bad Deciders than of Observers of good Deciders. Specifically, a Mann-Whitney U test on spending rates for those who spent anything across conditions yielded a marginally significant effect (Z = -1.73, p = .084), such that Observers who spent anything to punish a bad Decider tended to spend less than those who spent anything to compensate a good Decider (M_Bad Person Injustice_ = $9.57, M_Good Person Injustice_ = $13.40). Taken together, we replicate our previous results for the Good Person *Injustice* condition such that many Observers choose to spend something and they do so in relatively small amounts. However, we now see that even fewer Observers choose to punish the bad Decider, and those who do, do so in relatively small amounts. In other words, because participants in the *Bad Person Injustice* condition were not given the possibility of sanctioning the bad Decider to a sufficient degree, they instead mostly chose to do nothing.

Taking the results of Studies 2a and 2b together, we return to our key predictions that the reason individuals readily compensate innocent victims of harm to small degrees but rarely punish ill-deserving beneficiaries of gains is that the threshold for restoring justice differs across the two injustice types. It is our contention that for the former, justice is restored by providing some small amount of compensation, which signifies that a person has done a sufficiently good deed, but for the latter, justice is restored only by fully removing any ill-gotten gains, to deter future bad behavior. However, Studies 1, 2a, and 2b do not allow us to make this claim definitively. Consequently, we conducted the final experiment to better understand the psychological underpinnings of these decisions regarding justice restoration.

## Study 3

To provide greater empirical evidence that small compensations are sufficient to compensate illegitimate losses, but only large punishments are sufficient to punish illegitimate gains, we experimentally manipulated the amount of compensation given or punishment bestowed by the Observer in the same type of context as Study 1. By asking new participants to evaluate the now experimentally varied actions of this Observer, we are able to understand how people believe justice can be restored across these two types of injustices, and, critically, why.

### Participants

360 participants (Median Age = 34; 46.9% female, 52.8% male, 0.3% unknown gender) were recruited from the Amazon Mechanical Turk (mTurk) online panel and paid $1.00 for completion of the study. Of those, 12 sets of responses came from participants who shared the same IP address (e.g. two completions from a single IP address). To avoid the possibility that individuals completed the study more than once, all such completions were excluded from analyses. Additionally, five participants completed the experiment faster than would be possible had they watched all instructional videos (all five completed the study faster than 4 minutes, when the videos totaled about 5 minutes in the condition with the shortest set of videos) and were excluded from the study. This resulted in usable data from 343 participants.

### Procedure

Unlike Study 1, where participants actually played the third-party observer dictator game, in this study, participants instead watched a series of videos explaining all of the actions in the game, including a final spending decision by the Observer. Because the behaviors of interest from Study 1 occurred in the *Injustice* conditions, in the present study, we only include the two *Injustice* conditions: *Bad Person Injustice* (Decider gains $25) and Good *Person Injustice* (Decider loses $25). Specifically, participants were informed that they would learn about the actual behaviors of participants from a different study. They first watched an approximately 3 minute and 30 second long video describing the set-up of the game, the random selection process of role assignment, and the five rounds of play. For participants in the *Bad Person Injustice* condition, this video showed the Decider keeping all $20 across each of the five rounds of play. They learned that this resulted in allocations for the three players of: Decider = $100, Receiver = $0, Observer = $50. For participants in the Good *Person Injustice* condition, this video showed the Decider sharing half of his or her allocation ($10) across each of the five rounds of play. They learned that this resulted in allocations for the three players of: Decider = $50, Receiver = $50, Observer = $50. Participants then answered three attention check measures designed to ensure that they carefully watched and understood the video. Next, all participants watched an approximately 40 second video explaining the injustice. For participants in the *Bad Person Injustice* condition, the video explained that the Decider was randomly selected to receive an extra $25, bringing his or her total to $125. For participants in the *Good Person Injustice* condition, the video explained that the Decider was randomly selected to lose $25, bringing his or her total to $25. Participants then answered two attention check measures designed to ensure that they carefully watched and understood the video. Next, participants watched an approximately 1 minute and 30 second video explaining that the Observer was either given the chance to take money from the Decider (*Bad Person Injustice*) at a rate of $4 taken away for every $1 spent or give money to the Receiver (*Good Person Injustice*) at a rate of $1 given for every $1 spent. Following this video, participants were randomly assigned to one of seven *Amount Spent* conditions: $0, $5, $10, $15, $20, $25, and $32. These amounts were chosen to reflect the most common decisions undertaken by participants in Study 1. Of note, $32 was included because it would be the minimum amount needed to remove all moneys from the Decider ($32 x 4 = $126). Although this is a strange sum to give to the Decider in the Good Person condition, we included it to maintain the fully crossed experimental design.

Participants in both *Injustice Type* conditions were told that the Observer spent the corresponding amount of money and what that meant for the final allocations for all participants. Table 2 summarizes what participants saw in each condition. Note that because this was a fully between-subjects experiment, participants were only exposed to one of these allocations. For example, a participant in the *Bad Person Injustice + $10* condition learned that the Observer spent $10, resulting in a final allocation of $85 for the Decider, $40 for the Observer, and $0 for the Receiver.

**Table 2:** Summary of final payouts in Study 3.

Injustice Type Condition	Amount Spent	Decider	Observer	Receiver
Bad Person Injustice	$0	$125	$50	$0
	$5	$105	$45	$0
	$10	$85	$40	$0
	$15	$65	$35	$0
	$20	$45	$30	$0
	$25	$25	$25	$0
	$32	$0	$18	$0
Good Person Injustice	$0	$25	$50	$50
	$5	$30	$45	$50
	$10	$35	$40	$50
	$15	$40	$35	$50
	$20	$45	$30	$50
	$25	$50	$25	$50
	$32	$58	$18	$50

Next, participants answered a series of questions designed to ascertain the extent to which they believed justice had been restored and why. Specifically, to test our hypothesis that only small amounts of compensation are sufficient to restore justice when a bad thing befalls a good person, but that large amounts of punishment are necessary to restore justice when a good thing occurs to a bad person, participants were asked: “How fair is the final amount of money the Decider has?” on a 7-point scale anchored with 1 = “Not at all fair” and 7 = “Extremely fair”. To test our prediction that the smaller and larger amounts of compensation/punishment are associated with perceptions of having engaged in morally sufficient behavior, we asked participants, “To what extent has the Observer done something good?” on a 7-point scale anchored with 1 = “Not at all” and 7 = “Completely”; and “How good of a person is the Observer?” on a 7-point scale anchored with 1 = “Not at all a good person” and 7 = “Very much a good person”. To test our hypothesis that justice restoration for the bad person is based on a desire for deterrence of future bad behavior, participants indicated, “Given how much the Observer spent, how likely is the Decider to act the same away again if he or she played this study again?” on a 7-point scale anchored with 1 = “Not at all likely” and 7 = “Extremely likely”. Finally, we also asked participants “How likely would you have been to spend the same amount of money as the Observer spent?” on a 7-point scale anchored with 1 = “Not at all likely” and 7 = “Extremely likely” and to describe what they would have done had they been the Observer in an open ended text box. The two questions about what participants themselves would have done yielded little in terms of an understanding of how and why people believe that justice is differentially restored across the two conditions and so will not be discussed further. However, all data are freely available, as mentioned in the author note. Finally, the participants provided some basic demographic information about themselves.

### Results

Ten participants answered at least two of the attention check questions incorrectly and are thus omitted from all analyses. Including these participants (or additionally excluding the 51 participants who answered one of the attention check questions incorrectly) does not meaningfully change the results of our analyses.

**Fig 5:**
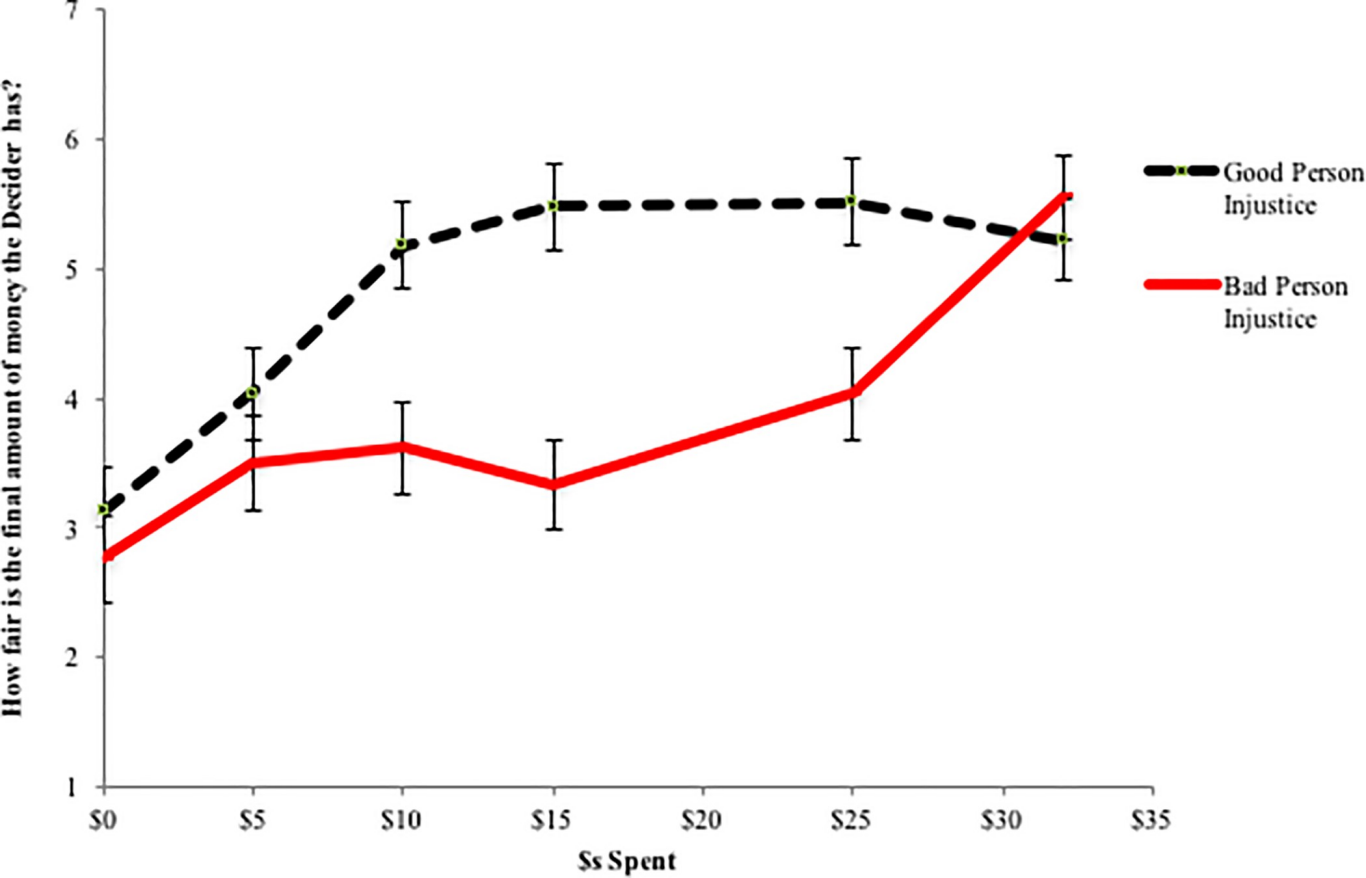
Study 3 perceptions of fairness by condition. Error bars represent standard errors.

How much compensation/punishment is required for justice to be restored across the two types of injustices? The most direct assessment of justice restoration is the extent to which, given the Observer’s actions, the Decider’s final allocation is considered to be fair. As Fig 5 clearly shows, and as confirmed by the 2 (Person Type) x 7 (Amount Spent) between subjects ANOVA on perceptions of fairness (interaction: *F*(5, 321) = 3.75, *p* = .003), the amount necessary for complete justice restoration depends on the type of injustice that transpired. When a bad event befalls a good person (*Good Person Injustice*), once some minimal amount of compensation is provided ($10, in this case), additional compensation does not result in greater assessments of fairness. That is, it is just as fair that the Decider ultimately received $10, $15, $20, $25, or $32 (all pair-wise comparisons *p* > .5). This result is consistent with the findings of Study 1; to restore an injustice that transpires against a good person, it is just as good to give a little as it is to give a lot.

In contrast, the pattern of results is quite different when the injustice is one where a bad person benefits (*Bad Person Injustice*). As can be seen in the same figure, perceived fairness for the Decider’s final allocation is only increased when most or all of his or her money is taken away ($25 or $32 spent). That is, as compared to $0 spent to punish the Decider, only $25 (*F*(1, 321) = 6.86, *p* = .009) and $32 (*F*(1, 321) = 35.19, *p* < .001) result in an increased perception of fairness (all other comparisons to $0, *p* > .1). In other words, small punishments are insufficient to restore justice; only punishments that remove all or most of the Deciders allocation result in justice restoration. Moreover, when all of the Bad Person Decider’s funds are taken away ($32 spent), justice is perceived to be as great as when the Good Person Decider is compensated as little as $10 (*F*(1, 321) = .61, *ns*). This, again, is consistent with the findings of Study 1, and further corroborates our interpretation of the Study 1 findings that if the only effective way to restore justice is to strip a Decider of most or all of his or her ill-gotten gains, then spending small amounts of money doesn’t make sense. However, spending enough to restore justice is quite costly, explaining why few people in the *Bad Person Injustice* condition in Study 1 ultimately chose to act at all.

**Fig 6:**
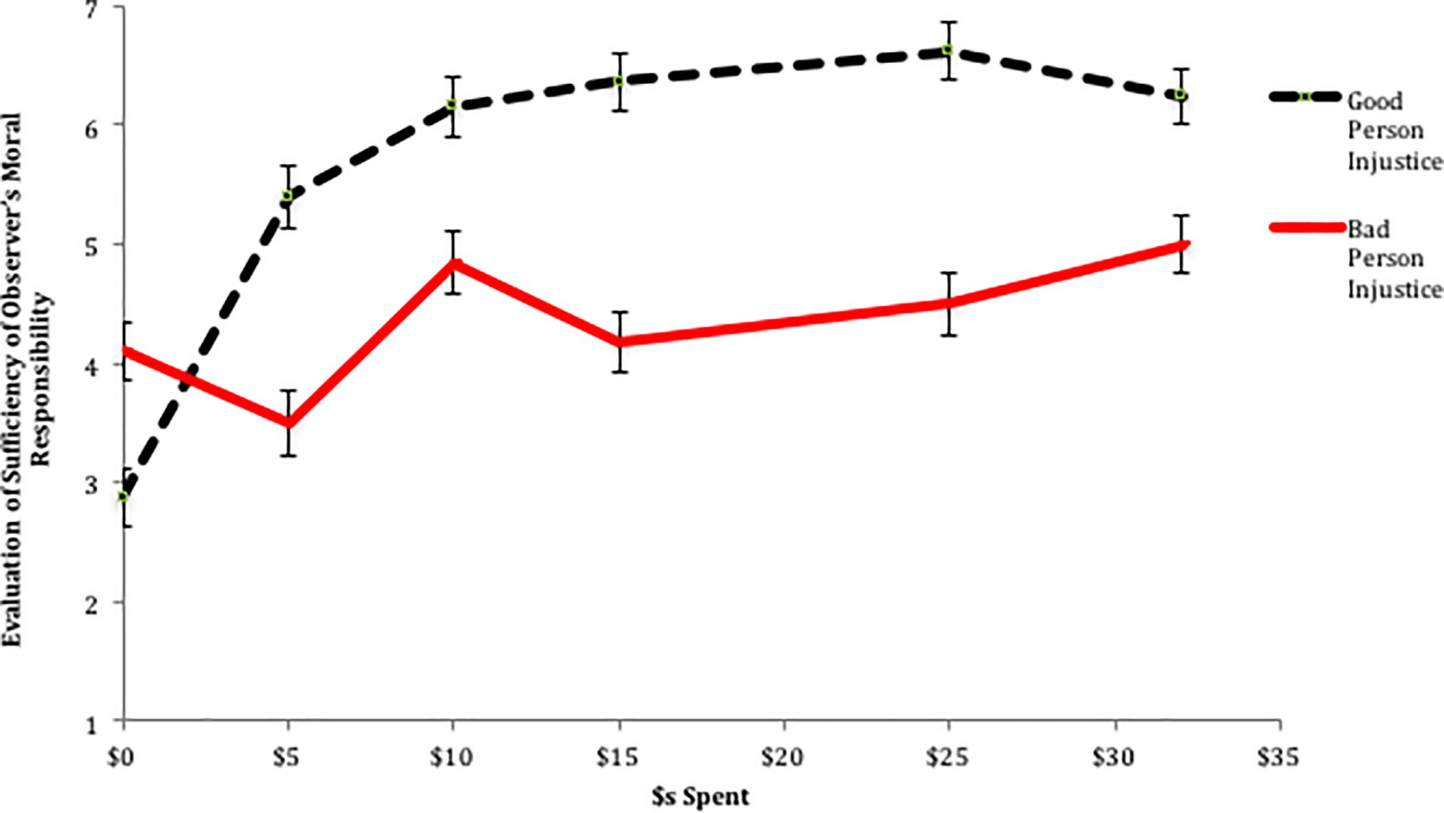
Study 3 evaluation of sufficiency of observer’s moral responsibility. Error bars represent standard errors.

Turning to why people hold such perceptions about what constitutes justice restoration, we consider two separate drivers: perceptions that the Observer’s actions are morally sufficient and the belief that the Decider will repeat his or her actions given the Observer’s behavior. For the former, we pool the responses to the two questions about the Observer: the extent to which he or she has done something good and the extent to which the Observer is a good person (r = .79, p < .001). This results in a single measure of how participants evaluated the Observer’s actions in terms of his or her moral soundness, with higher values indicating that the Observer has acted morally appropriately. As can be seen in Fig 6, and as confirmed by a similar ANOVA to the one above (interaction: *F*(5, 321) = 13.38, *p* < .001), perceptions of what constitutes sufficient moral behavior differ dramatically across injustice types. When a bad event befalls a good person (*Good Person Injustice*), as soon as some minimum amount of compensation is provided ($10, in this case), additional compensation does little to increase the perception that the Observer acted in a morally sound way. That is, doing nothing ($0 spent) is considered morally unacceptable. However, the Observer is seen to be just as good a person if he or she spent $10, $15, $25, or $32 (all pair-wise comparisons p > .13; spending $5 is seen as more morally acceptable than $0 (p < .001), but only slightly less than $10 (p < .01)). These results mirror the findings on perceived fairness of the compensation/punishment, providing evidence that the reason that justice is perceived to be restored with even small amounts of compensation is because a person is believed to have met their moral obligation by merely providing some (nominal) level of help.

Here, we would like to additionally point to the lack of a significant decrease in perceived morality at compensation levels above $15. If it was the case that participants were attuned to the equality between the Observer and the Decider and believed that the most just outcome was one in which the Observer compensated the Decider at an amount where both parties would have equal outcomes (i.e., the Observer and Decider end with approximately equal amounts), we should have observed a rise in perceived morality for the $10 or $15 conditions, and a drop in perceived morality for the conditions in which the Observer’s decision results in an inequality between the Observer and the Decider (with the Decider receiving more). That we do not observe this fall off suggests that, as in Study 2a, individuals are not focused on justice in terms of equality between the Observer and the Decider, but on justice in terms of restoring the Decider’s position.

We turn now to the results for individuals evaluating an Observer of a bad person who had received an unfair windfall. As with the fairness measure, the pattern of results is quite different when considering the amount spent to correct the injustice resulting from a benefit to a bad person (*Bad Person Injustice*). Two key findings stand out. First, regardless of the amount spent, the Observer’s actions are never seen as particularly morally sound, as compared to the Observer who compensates a victim (Main effect of Person type: *F*(1, 321) = 74.39, *p* < .001). That is, except for the case where no money is spent, the Observer is always perceived to be less morally sound when punishing the Bad Person than when compensating the Good Person. Second, and perhaps more telling, is that even though there is a main effect of *Amount Spent* within the *Bad Person Injustice* condition on the Observer’s perceived morality (*F*(5, 154) = 2.96, *p* = .01), there appears to be no systematic relationship between *Amount Spent* and perceptions of how morally sound the Observers actions were. That is, once the Observer spent even a little, they were seen to be just as moral as if they had spent a significant sum of money (examining all Bonferroni corrected pair-wise comparisons between spending amounts on perceptions of morality indicates only a significant difference between $5 and $32, a result that is relatively uninformative). Here, again, if participants were primarily concerned with the Observer and Decider receiving equal outcomes, we should have observed an increase in perceived morality in the $25 condition. That we do not, again, argues against a desire to simply achieve equality between the Observer and Decider.

More generally, it appears that regardless of what the Observer does, when punishing a bad person, his or her actions are seen as equally morally sound, but also, more importantly, less morally sound than a comparable Observer who compensates a good person. In other words, given that spending amount does not predict the degree to which one who punishes a bad person is morally sound, but perceptions of justice restoration are quite sensitive to amount spent, we lack evidence that when trying to restore justice by punishing, perceptions of moral soundness play a role. Thus, it cannot be the case that in Study 1, Observers who chose to punish did so because they wanted to feel as though they had done their moral duty. Instead, we propose that the other key variable, beliefs about the probability of changing the Deciders’ future behavior explains the differences in perceptions of justice restoration when punishing a bad person.

**Fig 7:**
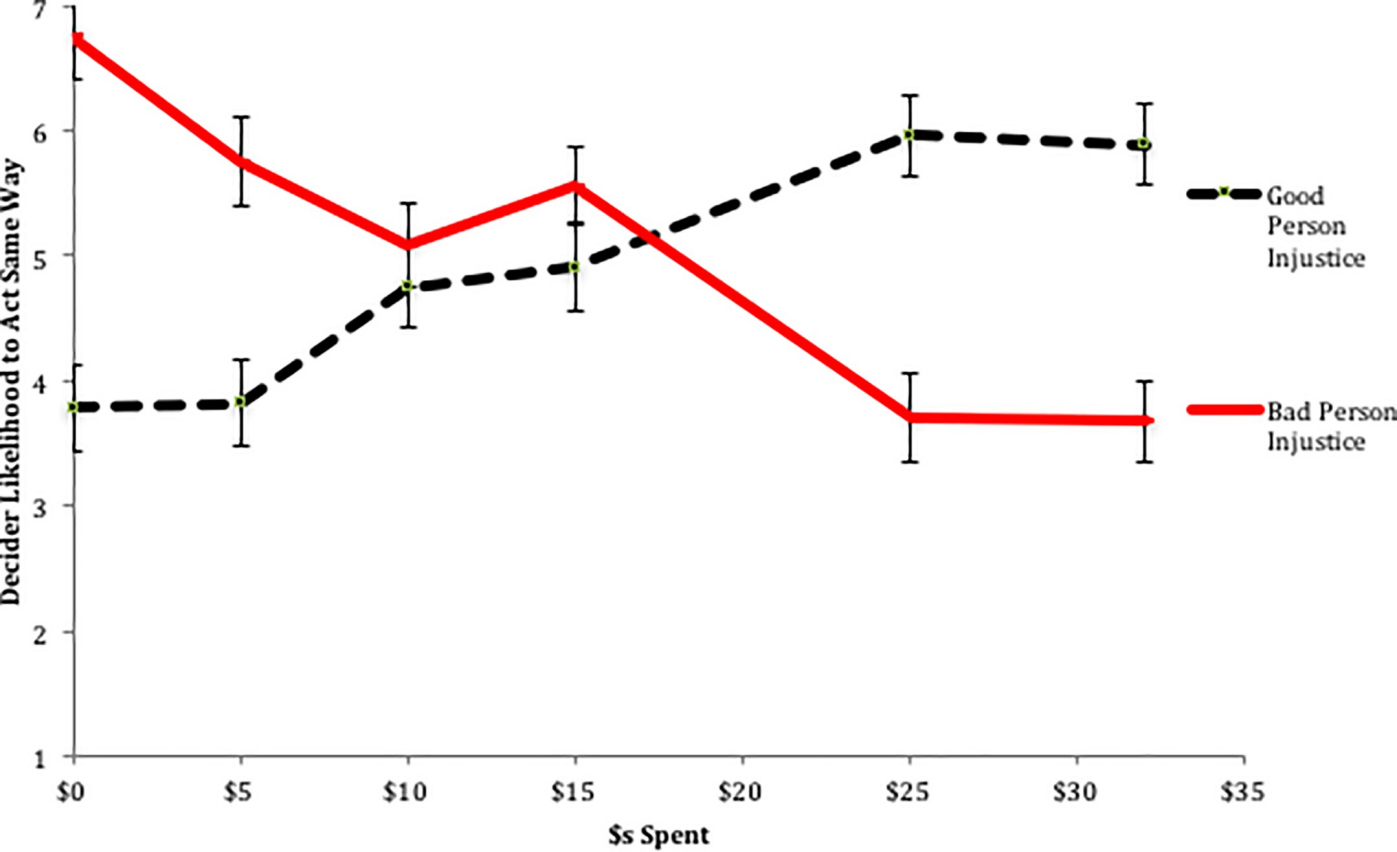
Study 3 Belief about decider’s likelihood to act the same way again. Error bars represent standard errors.

To test this possibility, we consider the degree to which participants believe that the Decider will act the same way again given the amount spent by the Observer. The argument here is that a good person is unlikely to continue to act as such if they experience negative events that are not corrected. Likewise, a bad person is unlikely to correct his or her bad ways if he or she is not sufficiently punished. As can be seen in Fig 7, and as confirmed by a similar ANOVA to the one above (interaction: *F*(5, 321) = 28.47, *p* < .001), regardless of whether an injustice occurred to a good or bad person, participants believe that greater spending will lead to more positive outcomes. When a good person has a bad event befall them, participants believe that as their compensation increases, so does the likelihood that they will continue to act as a good person (linear contrast, p < .001). Likewise, when a bad person experiences an unjust benefit, participants believe that as they are punished more, their likelihood to act selfishly again decreases (linear contrast, p < .001).

Though there is no divergence in the perceived future behavior of the Decider as a function of amount spent and injustice type, there is a significant difference in the extent to which this dimension is concordant with beliefs about justice restoration. Specifically, when a bad event befalls a good person, participants believe that the more that is spent to compensate this person, the more they are likely to act fairly again in the future. For instance, they believe that spending $25 will lead the person to act more fairly in the future than spending $15 (F(1, 321) = 3.44, p = .07) or $10 (F(1, 321) = 5.83, p = .02). Though this may well be true, as we saw above, there is no such relationship between amount spent and perceptions of justice restoration. That is, though there is an increase in perceptions of how likely the good person is to act fairly again as the amount spent to compensate them increases, there is no corresponding relationship in perception of justice restoration, suggesting that for injustices of this type, perceptions of how likely the actor is to act the same way again cannot explain responses to justice restoration.

Anticipated likelihood of future bad behavior can, however, explain responses to justice restoration for injustices that occur to bad individuals. As described above, when a bad actor benefits in some way, only near complete punishments (e.g. $25 or $32 spent) are considered to be complete justice restoration. Here, we see that this is the exact pattern observed for perceptions of how amount spent will result in the bad actor behaving the same way; there are no differences in perceptions of how likely the bad actor is to act the same way when either $5, $10, or $15 are spent (all pair-wise comparisons, p > .25), but there is a large drop in the perceived likelihood that the bad actor will again act selfishly when most or all of his or her money is taken away as compared to $15, the largest non-near complete amount spent ($25 spent: F(1, 321) = 22.72, p < .001; or $32 spent: F(1, 321) = 21.88, p < .001). Said otherwise, perceptions of justice restoration are concordant with the degree to which it is believed that a bad actor will not act badly again. Taken together with the results of Study 1, these results are consistent with the possibility that participants who chose to punish the bad person who received an illegitimate gain (who almost all did so by completely removing the Deciders’ ill-gotten gains) did so because they believed that by doing so, they were deterring future bad behavior, and only the deterrence of future bad behavior would count towards justice restoration.

A few points are worth mentioning in relation to this study. First, because we do not directly observe the motivations of participants in Study 1, an alternative interpretation of the present results is that participants in this study may have been judging the anticipated efficacy of the behavior that they observed, rather than the evaluating the underlying motivation that could have been driving the behavior they observed. That is, participants may have inferred the observer’s motivation was deterrence, or they could have inferred that deterrence was the result of the observer’s actions, without necessarily knowing (or considering) the underlying motivations of the observer’s behavior. Others have noted this distinction and have labelled them “psychological” and “biological” altruism, respectively [50]. Future work could provide clearer evidence which form of altruism drives compensatory vs. punitive behavior towards victims of unfair losses and beneficiaries of unfair gains.

## General discussion

In the face of injustice, even when the injustice is random in nature, people often feel an overwhelming need to personally rectify the wrongs they witness [2]. However, the extent to which they will do so is quite different depending on the nature of the injustice. When people witness an otherwise good person experience an ill-deserved hardship that is not of their own causing, they feel compelled to help that person, but only in small amounts. It appears that even small acts of compensation are seen as sufficient to convince individuals that they have fulfilled their moral obligations and have restored justice. In contrast, when people see a bad person receiving an illegitimate benefit, they are shy to act, but when they do, they act completely. For them, it is not enough to just slap a bad person on the wrist; the punishment must be severe enough to deter future bad behavior. As it happens, the level of punishment necessary to deter future bad behavior is also one that nearly or completely wipes out any of the ill-gotten gains.

As far as we know, we are some of the first to explicitly explore people’s differential responses to objectively equivalent injustices in which good persons obtain negative outcomes and bad persons obtain positive outcomes. In so doing, our findings differ from those found in prior work that has identified the need to provide “just desserts,” rather than a desire to deter future bad behavior, as a primary driver of punishment behavior [7]. Rather, we show that the desire for deterrence may not be completely irrelevant to peoples’ punishment decisions. Instead, perhaps whereas a desire for just desserts will drive punishment behavior when a bad actor causes their good outcomes, a desire for deterrence drives punishment behavior when a bad actor randomly receives a good outcome. Specifically, in our “no injustice” condition in Study 1, when the bad actor benefitted directly from his or her own actions, participants did punish the actor, but only in small amounts, which is consistent with a “just desserts” perspective. However, when the bad actor randomly received a good outcome, participants’ behaviors were consistent with deterrence: if they chose to punish, they did so completely. Perhaps, then, deterrence is seen as needed primarily when bad actors are seemingly “rewarded” by the universe. After all, if the universe “rewards” bad actors, it is reasonable to believe that bad actors will continue acting poorly [2]. It is only then that punishment need be so severe that future bad behavior will be discouraged.

We believe that our findings are important for our understanding of psychological and behavioral responses to injustice in two ways. First, we document that injustices are broader than those causally related to individuals’ behavior; random outcomes can also be seen as unjust. These injustices are responded to with small acts of kindness or sizeable acts of punishment, for good actors obtaining negative outcomes and bad actors obtaining positive outcomes, respectively. Given this, in organizations, the moral character of employees becomes very important. Even if outcomes are not caused by an employee’s actions, if s/he is seen as achieving an outcome that is not commensurate with his/her moral character, others may react in ways that can influence effective workplace performance. For example, if a known womanizer wins a company-wide lottery for a coveted prize, our project suggests that most individuals will see that outcome as unfair and undeserved. In addition, while the majority of individuals might not respond with any action, some individuals could be driven by a desire to restore justice to engage in extreme acts of punishment designed to remove the bad actor’s good outcomes. While it’s unclear from the present work what those actions might be, one could surmise that whatever they are, they are likely to have negative effects on group performance or team functioning. Justice-restorative behavior, in this case, could include not cooperating with the “unfairly” rewarded bad actor to sabotaging their work by actively withholding necessary information or providing incorrect information. In this way, our findings provide further justification for Robert Sutton’s “No asshole rule;” seeing bad actors obtain good outcomes, even if those outcomes are due to luck alone, could be corrosive to the social order or, in the context of organizations, the work climate [51].

We also found that good actors who received bad outcomes were also perceived to be victims of injustice. Yet, participants’ willingness to provide compensation, while high in frequency (i.e., many elected to provide some compensation), were low in amount (i.e., they did not give very much money). This raises the question of how victims of random acts experience their outcomes and others’ responses to those outcomes. Do they too experience random negative outcomes as unjust? If so, what do they expect in terms of responses from others? For example, victims of natural disasters might believe that while the disaster may have been random, they did not deserve to suffer and therefore should receive significant aid from others. And, while observers might concur that the victims did not deserve their outcome, they are unlikely to engage in appropriately compensatory behavior in that the amount of aid that they are willing to provide may not be sufficient to assuage victims’ sense of injustice, thereby leading to the belief by victims that they are not valued or respected by others. Here, too, one could imagine that the social and organization implications of these unmet expectations on the part of victims could be detrimental to group functioning; if workers do not feel as though they have received the respect and value that they believe is due to them, they will respond by reducing their commitment to the group [52, 53]. This possibility suggests that there might be a mismatch between what observers and victims consider to be fair, and that this mismatch could have negative effects on group performance or functioning.

A limitation of our work is that we did not vary the degree of loss suffered by the good person. Specifically, in all of our studies, the good person lost some portion of their allocation, but never lost *all* of their allocation. We avoided this scenario because there is no parallel gain that a bad person could experience that would be equal to a complete loss. Even if we matched the absolute dollars lost and gained, it is psychologically different to have nothing than just having less, and there is no parallel on the gain side [54]. That said, future work could explore if people choose to compensate victims of total loss differently than those of partial loss. For instance, perhaps the fact that victims lost everything they had (say, in a hurricane), individuals feel a greater desire to provide larger compensation than when those same victims still retain some of their possessions.

We also want to be clear that we anticipate decisions about compensation and punishment to be multi-determined. We do not mean to claim that the mechanisms that we find here–that of fulfilling moral obligation when providing compensation, but of deterrence when meting out punishment–are the only ones that drive justice-restorative behavior. For example, other work shows that cognitive reflection influences the propensity to engage in antisocial behavior [55], and it is possible that an individual’s propensity to engage in cognitive reflection might moderate the present results. However, we are able to rule out at least two potential alternative explanations–inequality aversion (Study 2a) and concern for punishment extremity (Study 2b)–and believe that the present evidence supports the possibility that decisions to compensate unjust victims is, at least in part, driven by concerns about fulfilling moral obligations, while the decisions to punish unjustly rewarded transgressors is, at least in part, driven by concerns about future bad behavior. We hope that the present work prompts thinking about the variety of psychological motivations that play a role in individuals’ responses to injustice, particularly as observers.

In sum, injustice occurs when actors receive outcomes that are incommensurate with their moral character [56]. Bad actors ought to receive negative outcomes, and good actors ought to receive positive outcomes. Yet, in social and organizational life, this is often not the case. We show that even when the actors are not directly responsible for their outcomes, injustice is still perceived to have occurred. Notably, how outside observers respond to these two types of injustice differ greatly; they are more than willing to compensate victims of random negative events, but do so only in small amounts because they perceive their moral obligation to have been fulfilled with compensation of any amount. Moreover, they are reluctant to punish bad actors who have randomly obtained positive outcomes, but once they choose to engage in punishment, the punishment is complete; they remove the positive outcome in its entirety because only complete punishment is believed to be sufficient to change a bad actor’s behavior. In this way, our work adds to research documenting how important justice is to individuals and for social functioning.

## Supporting information

S1 Appendix Screenshots of Study 1. (DOCX)

S2 Appendix Supplemental study description and results. (DOCX)
