# Supplementary material for: Compensate a little, but punish a lot: Asymmetric routes to restoring justice
Source: PLoS One. 2019 Jan 10;14(1):e0210676. doi: 10.1371/journal.pone.0210676 (PMC6328196; doi:10.1371/journal.pone.0210676)

Figure A: Waiting Room


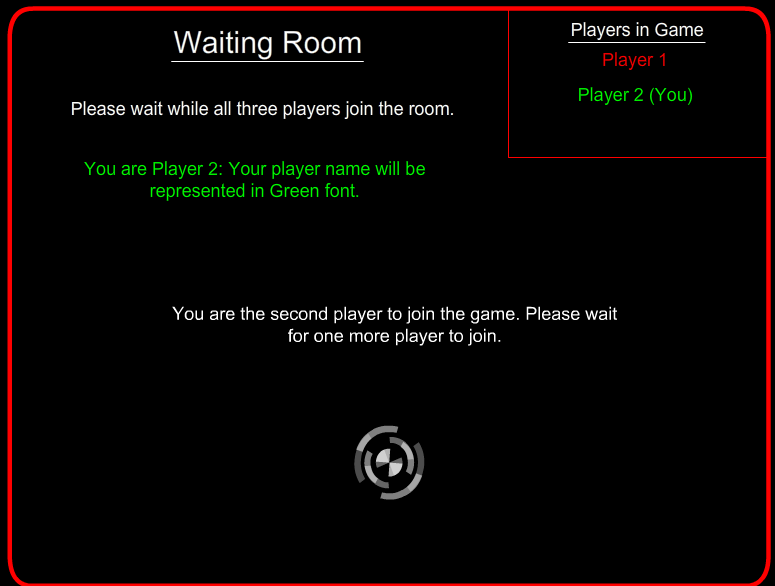


Figure B - Practice Round 1 Results


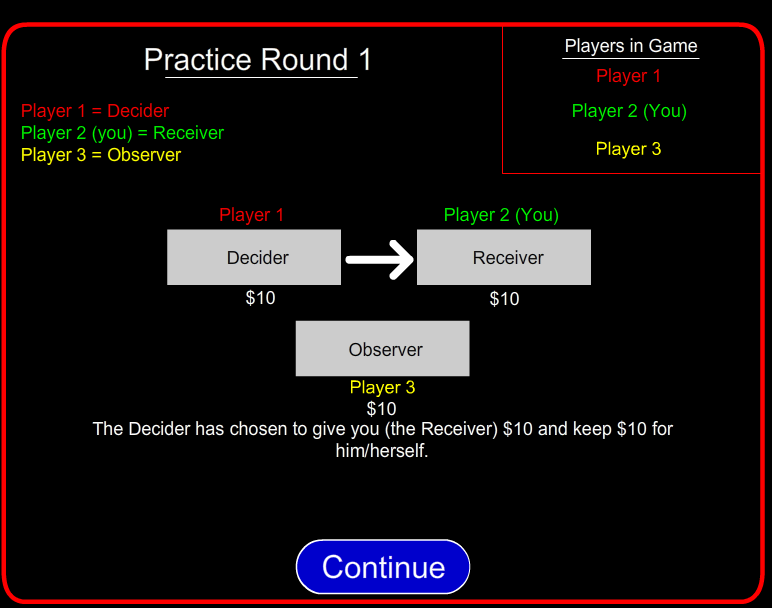


Figure C – Practice Round 3


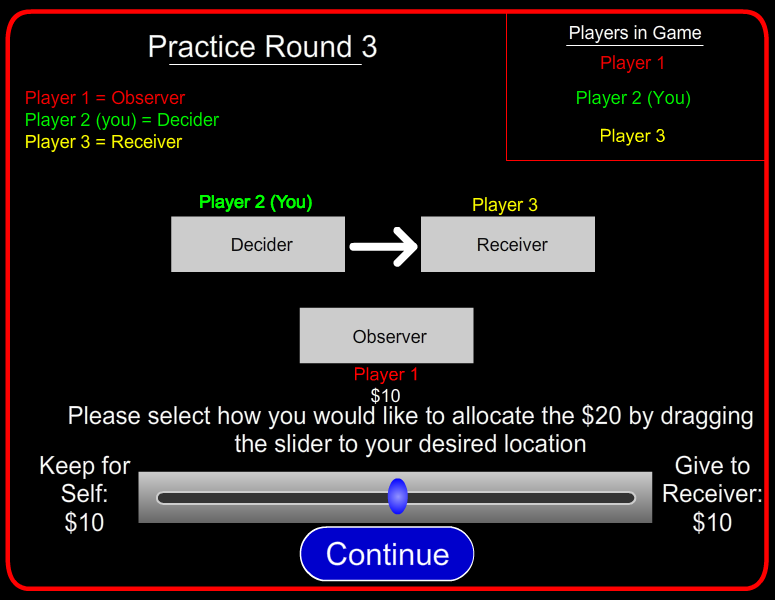


Figure D – Summary Screen Following “Bad” Person Winning Bonus $25


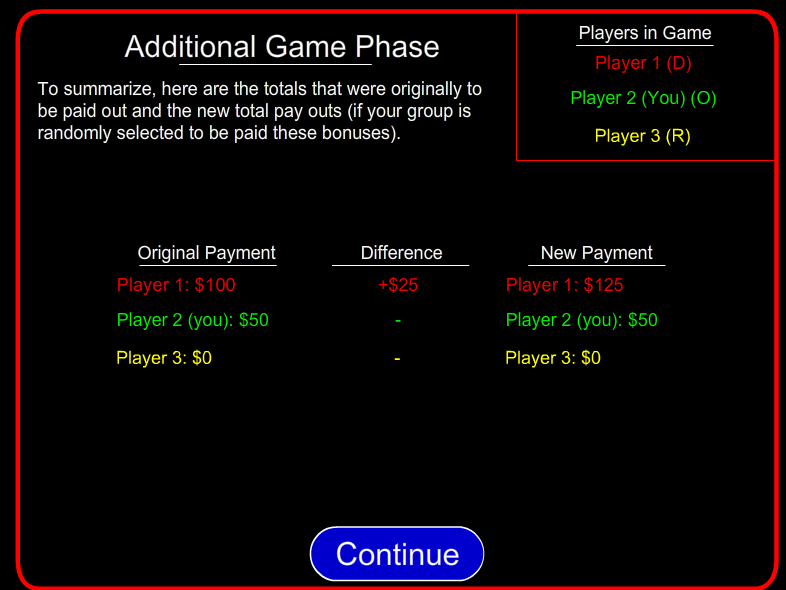


Figure E – Decision Screen For *Bad Person - Injustice* Condition


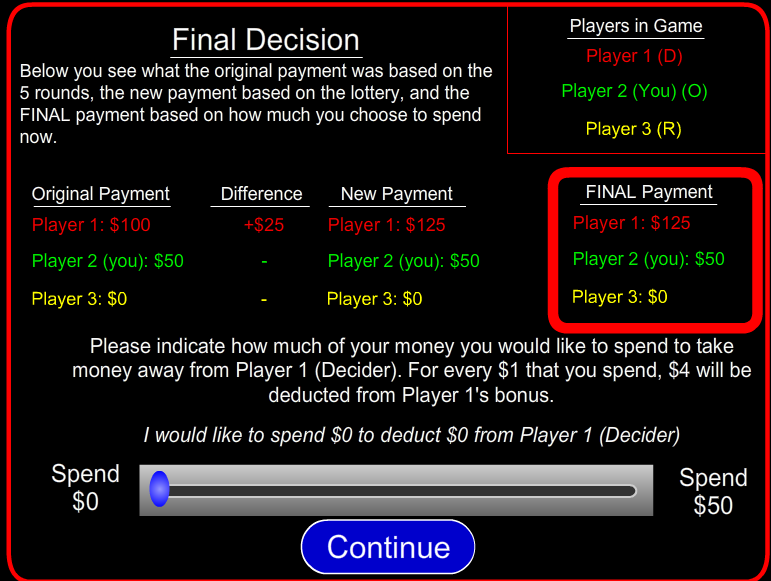

Supplement: S1 Appendix — (DOCX) [file pone.0210676.s001.docx]
