# Supplementary material for: Compensate a little, but punish a lot: Asymmetric routes to restoring justice
Source: PLoS One. 2019 Jan 10;14(1):e0210676. doi: 10.1371/journal.pone.0210676 (PMC6328196; doi:10.1371/journal.pone.0210676)
Supplement: S2 Appendix — (DOCX) [file pone.0210676.s002.docx]

The present study was a replication of the Study 1 with the following key differences. First, there were only two between-subjects conditions. Specifically, only the two conditions where an injustice transpired (*Good Person Injustice* and *Bad Person Injustice*) were included. Second, rather than compensating participant actions with a lottery, all payment amounts were reduced by an 100x to allow for direct individual compensation. So rather than being able to earn, say $50 as the observer in expectation (if the participant won the lottery and spend none of his/her money to compensate or punish the actor), the observer was guaranteed a $0.50 payout under the same conditions. Third, we a series of exploratory measures were added to attempt to understand some alternative aspects of the decisions made in Study 1. Specifically, after learning that the Decider either lost $0.25 (*Good Person Injustice*) or won a bonus $0.25 (*Bad Person Injustice*), participants answered the following questions:

1. Independent of their feelings about the Decider, how bad (good) is it lose (win) $0.25 in this game (-4 = Extremely Bad, 0 = Neutral, +4 = Extremely Good)
2. Thinking about the actual game that just took place, how fair was what just happened to Player 1 (Decider) (winning a bonus $0.25/losing $0.25)? (-4 = Extremely Unfair, 0 = Neutral, +4 = Extremely Fair)
3. How likely is it that Player 1 (Decider) will change because of the event that happened to him/her? (-4 Player 1 is likely to become a WORSE person, 0 = Player 1 is unlikely to change, +4 = Player 1 is likely to become a BETTER person)
4. To what extent did what happened to Player 1 result in any negative emotions (1 = Not at all, 7 = To a Great Extent)
5. Allocate 100% across 6 possible negative emotions that participants may have experienced in response to what happened to Player 1. The emotions were Angry, Upset, Sad, Tense, Disgusted, Other Negative Emotions.
6. To what extent did what happened to Player 1 result in any positive emotions (1 = Not at all, 7 = To a Great Extent)
7. Allocate 100% across 6 possible positive emotions that participants may have experienced in response to what happened to Player 1. The emotions were Happy, Calm, Serene, Elated, Glad, Other Positive Emotions.

Other than this, the study was identical to the Study 1.

Participants:

300 participants were recruited from the Amazon Mechanical Turk (mTurk) online panel and paid a $1.00 show up fee regardless of their actions during the study. Of those, 4 participants did not actually complete the study, but rather submitted an erroneous completion code and did not complete any study materials or measures. Accordingly, this resulted in usable data from 296 participants (Median Age = 32, 54% female)

Results:

Manipulation Checks

We next confirm that the Decider was, in fact, perceived as being a good or bad person depending on their actions in the two Injustice Type conditions. They were: the good person was perceived to be far better (M = 3.19) than the bad person (-2.33; t(294) = 37.19, p < .001). As a second manipulation check, we test to see if participants perceived the event (winning/losing $0.25) to be relatively good or bad depending on condition. Again, they did. Losing $0.25 was seen to be quite bad (M = -2.48) as compared to the good event (M = 2.46; t(294) = 24.27, p < .001).

Primary Decision

We first analyze the decision to punish vs compensate by assessing differences spending rates across all conditions. Because spending rates are highly non-normally distributed (Shapiro-Wilk test of normality(296) = .72, p < .001), we again cannot use simple parametric analyses such as t-tests. Instead we assess differences in spending rates with non-parametric tests. For presentation purposes, we report means rather than medians, but use Mann-Whitney tests for pair-wise comparisons. Specifically, we find that participants in the *Good Person Injustice* condition spent slightly more (M = $0.091) than participants in the *Bad Person Injustice* condition (M = $0.072; Mann-Whitney U = 7526.5, Z = 4.98, p < .001)

The results, however, are quite different when considering the question of how often participants chose to spend any amount of their own money as well as the quantity they chose to spend. A logistic regression predicting the likelihood of giving any amount of money as a function of injustice type yielded a significant result (B = -.85, SE = .18, p < .001). Specifically, whereas 68.4% of participants in the *Good Person Injustice* condition spent any money to compensate the victim, only 29.9% of participants in the *Bad Person Injustice* condition spent any money to punish the bad person. Of note, this effect holds even when controlling for both perceptions of how good or bad the person and how good or bad the event was.

However, despite the large difference in the numbers of people who spent anything to compensate or punish, the rates at which these people did so was exactly opposite. Specifically, participants who spent any money, spent considerably more to punish a bad person who had a good action befall them (M = $.24) as compared to compensate a good person who had a bad action befall them (M = $.13 Mann-Whitney U = 1430.5, Z = 3.49, p < .001).

All other exploratory results are summarized in SOM Table 1 below.

Of note, none of the exploratory measures collected provided any process evidence for giving rates or amounts. That is, mediation models including any of the variables collected did not result in significant mediation of giving rates or amounts suggesting that though some measures did differ by condition, they were epiphenomenal to the main results.

Table 1: Summary of Results by Condition

|  | All Participants | | Participants Paying Anything | |
| --- | --- | --- | --- | --- |
| Variable | Bad Person Injustice | Good Person Injustice | Bad Person Injustice | Good Person Injustice |
| Manipulation Checks |  |  |  |  |
| Person G/B | -2.33 (0.12)* | 3.19 (0.09) | -2.93 (0.17)* | 3.33 (0.09) |
| Abs(Person G/B) | 2.36 (0.12)* | 3.19 (0.09) | 2.93 (0.17)* | 3.33 (0.09) |
| Event G/B | 2.46 (0.17)* | -2.48 (0.12) | 2.58 (0.32)* | -2.42 (0.13) |
| Abs(Event G/B) | 2.90 (0.11)* | 2.59 (0.10) | 3.14 (0.17)* | 2.50 (0.12) |
|  |  |  |  |  |
| General Measures |  |  |  |  |
| Fair | -1.53 (0.22)* | -3.15 (0.12) | -2.30 (0.37)* | -3.28 (0.13) |
| Person Change | -1.28 (0.14)* | -0.57 (0.12) | -1.42 (0.26)* | -0.62 (0.13) |
|  |  |  |  |  |
| Negative Emotions |  |  |  |  |
| General Negative Emotions | 4.51 (0.15) | 4.61 (0.13) | 5.16 (0.24) | 4.78 (0.16) |
| Angry | 21.82 (1.91)* | 14.53 (1.26) | 23.74 (3.35)* | 15.21 (1.6) |
| Upset | 20.67 (1.92) | 20.59 (1.51) | 19.49 (3.5) | 22.84 (1.83) |
| Sad | 13.25 (1.9)* | 26.11 (2.23) | 15.07 (3.75)* | 26.87 (2.7) |
| Tense | 9.93 (1.67) | 11.38 (1.56) | 6.84 (2.3) | 10.1 (1.57) |
| Disgust | 18.71 (1.89)* | 12.2 (1.38) | 25.63 (3.95)* | 11.95 (1.62) |
| Other Negative Emotions | 15.62 (2.27) | 15.2 (1.99) | 9.23 (2.68) | 13.04 (2.1) |
| Weighted Angry | 118.74 (11.37)* | 74.53 (7.18) | 132.79 (19.83)* | 81.65 (9.61) |
| Weighted Upset | 97.9 (9.2) | 103.12 (8.06) | 108.95 (20.92) | 116.58 (9.78) |
| Weighted Sad | 48.24 (6.14)* | 113.93 (9.81) | 57.05 (12.62)* | 118.6 (11.71) |
| Weighted Tense | 41.41 (6.55) | 47.7 (6.06) | 35.95 (11.65) | 46.36 (6.79) |
| Weighted Disgust | 93.6 (9.74)* | 63.36 (7.57) | 137.4 (21.53)* | 64.14 (8.96) |
| Weighted Other Neg Emotions | 50.81 (7.23) | 58.55 (7.21) | 44.14 (12.66) | 50.56 (7.14) |
|  |  |  |  |  |
| Positive Emotions |  |  |  |  |
| General Positive Emotions | 1.69 (0.1) | 1.74 (0.11) | 1.44 (0.16) | 1.61 (0.11) |
| Happy | 2.90 (0.77)* | 8.08 (1.66) | 4.09 (1.41) | 4.34 (1.01) |
| Calm | 47.41 (3.68)* | 38.61 (3.45) | 48.02 (6.76) | 39.41 (4.22) |
| Serene | 7.52 (1.55) | 7.53 (1.55) | 7.7 (2.95) | 9.19 (2.16) |
| Elated | 2.35 (0.81) | 3.14 (0.89) | 2.47 (0.99) | 1.52 (0.5) |
| Glad | 5.97 (1.61) | 7.29 (1.5) | 4.91 (2.31) | 6.74 (1.84) |
| Other Positive Emotions | 33.85 (3.72) | 35.36 (3.58) | 32.81 (6.72) | 38.8 (4.39) |
| Weighted Happy | 8.91 (4.27)* | 28.35 (8.3) | 9.56 (5.67) | 8.86 (2.36) |
| Weighted Calm | 75.28 (7.56) | 59.38 (7.38) | 67.16 (11.48) | 63.02 (10.12) |
| Weighted Serene | 12.68 (3.04) | 13.08 (2.84) | 9.63 (3.82) | 14.77 (3.86) |
| Weighted Elated | 5.42 (2.87) | 7.16 (2.36) | 3 (1.26) | 3 (1.22) |
| Weighted Glad | 20.6 (7.35) | 15.42 (3.41) | 12.42 (8.54) | 12.8 (3.62) |
| Weighted Other Pos Emotions | 46.56 (6.08) | 50.3 (6.05) | 42.42 (9.71) | 58.13 (8.1) |
|  |  |  |  |  |
| Payment Behavior |  |  |  |  |
| % Paying Anything | 0.30 (0.04)* | 0.68 (0.04) | -- | -- |
| Payment $ | $0.072 (0.01)* | $0.091 (0.01) | $0.241 (0.02)* | $0.133 (0.01) |

Note—* denotes statistically significant difference at the .05 level within Participant Type columns. Weighted emotion measures are computed by multiplying the stated % of feeling given emotion by the stated amount of that emotion felt.
